# Left–right reversal of the testes within the scrotum: An extremely rare variant of testicular ectopia

**DOI:** 10.1002/iju5.12549

**Published:** 2022-10-28

**Authors:** Kenjiro Kishitani, Kenji Yoda, Satoru Taguchi, Hikaru Suyama, Hayato Hoshina, Yoshiyuki Akiyama, Yuta Yamada, Yusuke Sato, Daisuke Yamada, Haruki Kume

**Affiliations:** ^1^ Department of Urology, Graduate School of Medicine The University of Tokyo Tokyo Japan

**Keywords:** cryptorchidism, ectopic testis, male infertility, testicular ectopia, undescended testis

## Abstract

**Introduction:**

Testicular ectopia refers to abnormal positioning of testis, which includes a wide variety of variants. An ectopic testis is located off the normal path of male gonadal descent, unlike conventional undescended testis.

**Case presentation:**

A 37‐year‐old man presented with the complaint of a palpable lesion in the scrotum. Magnetic resonance imaging of the scrotum revealed bilateral testes on the respective opposite sides of the scrotum with bilateral spermatic cords crossing under the base of the penis. Accordingly, he was diagnosed as “left‐right reversal of the testes within the scrotum.” In retrospect, the “palpable” lesion was thought to be the spermatic cords crossing above the testes. Semen analysis identified deteriorated sperm motility, suggesting possible male infertility.

**Conclusion:**

This case of left–right reversal of the testes within the scrotum is probably a new variant of testicular ectopia that has never been reported.

**Keynote message**

Testicular ectopia refers to abnormal positioning of testis and includes a wide variety of variants. An ectopic testis is located off the path of normal descent, unlike conventional undescended testis. We report a case of left–right reversal of the testes within the scrotum, whereby bilateral testes were located on the respective opposite sides of the scrotum with bilateral spermatic cords crossing under the base of the penis. The present case might be a new variant of testicular ectopia that has never been reported.

## Introduction

Testicular ectopia is a generic term used to refer to the abnormal positioning of the testis. It accounts for approximately 10% of extrascrotal testes and is sometimes contrasted with undescended testis or cryptorchidism, the commonest genital malformation in boys.^1^, ^2^ The difference lies in the fact that an undescended testis is located on the normal path of male gonadal descent while an ectopic testis is not. Common variants of testicular ectopia include perineal ectopic testis (the most common variant), femoral ectopic testis, pubopenile ectopic testis, and transverse testicular ectopia, while a wide variety of other variants have been described in the literature.^1^ We herein report a case of left–right reversal of the testes within the scrotum, probably a new variant of testicular ectopia that has never been reported.

## Case presentation

A 37‐year‐old man who had a past medical history of proteinuria (renal biopsy revealed no obvious abnormality) and was otherwise healthy visited our department with the complaint of a palpable lesion in the scrotum. He was suspected having left spermatocele by ultrasonography at another clinic 8 years previously but had undergone no further examination. A physical examination of the external genitalia demonstrated no abnormal findings. Laboratory blood tests did not detect any abnormal values, including serum total testosterone (6.71 ng/mL).

**Fig. 1:**
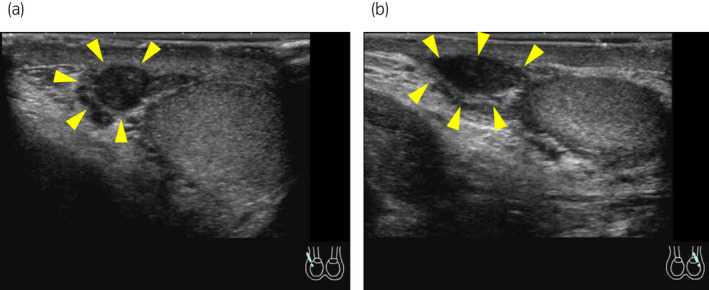
Ultrasonography of the scrotum (a, right; b, left) showed bilateral spindle‐shaped low echoic lesions located above the respective bilateral testes and epididymides (arrowheads), which had blood‐flow signals inside. It also detected normal but slightly small bilateral testes (a, right, 3.3 × 1.5 cm; b, left, 3.7 × 1.4 cm).

**Fig. 2:**
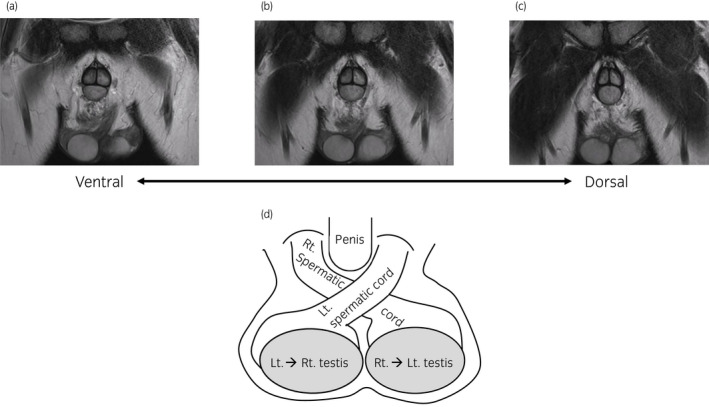
Magnetic resonance imaging of the scrotum (a–c, coronal section images from the ventral to the dorsal aspect) revealed that bilateral spermatic cords crossed under the base of penis and that the left spermatic cord was connected to the right (originally left) testis and vice versa. Overall, bilateral spermatic cords, epididymides, and testes were placed within the scrotum (d, a schematic illustration of the patient's external genitalia).

Ultrasonography of the scrotum showed bilateral spindle‐shaped low‐echoic lesions located above the respective bilateral testes and epididymides, which had blood‐flow signals inside. It also detected normal but slightly small bilateral testes (right, 3.3 × 1.5 cm; left, 3.7 × 1.4 cm) (Fig. 1). For further examination, the patient underwent magnetic resonance imaging of the scrotum, which revealed that bilateral spermatic cords crossed under the base of the penis and that the left spermatic cord was connected to the right (originally left) testis and vice versa. Overall, bilateral spermatic cords, epididymides, and testes were located within the scrotum (Fig. 2). Based on these findings, the patient was diagnosed as “left‐right reversal of the testes within the scrotum.” Ultrasonography and magnetic resonance imaging identified no other genitourinary abnormalities including the prostate, urinary bladder, and bilateral kidneys. In retrospect, the bilateral spindle‐shaped low‐echoic lesions detected by ultrasonography as well as the “palpable” lesion (the chief complaint of the patient) were considered to be the spermatic cords crossing above the testes and epididymides.

Screening semen analysis identified normal semen volume and sperm concentration and morphology but deteriorated sperm motility (both total and progressive), suggesting possible male infertility (Table 1). However, he did not wish further examination and/or treatment and therefore decided to discontinue visiting our department.

**Table 1:** Semen analysis results of the patient

Parameter	Value
Semen volume	3.0 mL
Semen pH	7.8
Sperm concentration	21 × 10^6^/mL
Sperm motility (total)	7.15%
Sperm motility (progressive)	0.41%
Sperm morphology	50% with normal morphology

## Discussion

The present report describes a case of left–right reversal of the testes within the scrotum, whereby each testis moves to the opposite side of the scrotum with bilateral spermatic cords crossing under the base of the penis. To the best of our knowledge, this condition has never been described in the literature and might thus be a new variant of testicular ectopia. The patient also presented with deteriorated sperm motility with slightly small testes, suggesting possible male infertility, although its association with his testicular ectopia was unclear.

Testicular ectopia refers to abnormal positioning of the testis and is distinguished from conventional undescended testis or cryptorchidism by the fact that an ectopic testis is located off the normal path of male gonadal descent.^1^, ^2^ Testicular ectopia includes a wide variety of variants, such as perineal ectopic testis (the most common variant), femoral ectopic testis, pubopenile ectopic testis, and transverse testicular ectopia.^1^ The last example is also known as “crossing testicular ectopia” and may thus resemble the present case. Transverse testicular ectopia is a rare form of testicular ectopia first described by von Lenhorsek in 1886,^3^ whereby both testes descend along the same inguinal route and ultimately lie on the same side of the scrotum.^4^, ^5^, ^6^ However, although a unilateral testis and spermatic cord moves across the midline *before* entering the inguinal canal in transverse testicular ectopia, bilateral testes and spermatic cords move across the midline *after* leaving the respective inguinal canals and cross each other in the present case, suggesting different underlying mechanisms.

The descent of testes through inguinal canals in the fetus occurs at 26 to 28 weeks as the gubernaculum pulls down the testes into the scrotum, after which the testes are fixed at the bottom of the scrotum at 35 to 40 weeks.^7^ Neither the physiology of normal testicular descent nor the cause of abnormal descent are clearly understood. In the present case, possible mechanisms include: (1) each testis descended into the opposite side of the scrotum after normally descending through each inguinal canal (at 26 to 28 weeks); or (2) bilateral testes became “twisted” within the scrotum after reaching the bottom of the scrotum normally (at 35 to 40 weeks). The latter seems more plausible if the scrotal septum can get twisted together with the testes, albeit unprovable.

Surgical correction is generally recommended for testicular ectopia as well as undescended testis, since it may optimize testicular function, facilitate diagnosis of testicular cancer, provide cosmetic benefits, and prevent complications such as hernia or torsion.^1^, ^8^ However, given that bilateral testes were located within the scrotum, an indication for surgical treatment might be controversial in the present case. Nevertheless, the patient should be cautiously followed up for cancer screening, although he wished to discontinue continuous checkup.

In summary, we report for the first time a case of left–right reversal of the testes within the scrotum, which is presumably a new variant of testicular ectopia.

## Author contributions

Kenjiro Kishitani: Conceptualization; data curation; formal analysis; writing – original draft. Kenji Yoda: Conceptualization; data curation; formal analysis; project administration; writing – review and editing. Satoru Taguchi: Conceptualization; data curation; formal analysis; project administration; writing – original draft. Hikaru Suyama: Data curation; writing – review and editing. Hayato Hoshina: Data curation; writing – review and editing. Yoshiyuki Akiyama: Data curation; writing – review and editing. Yuta Yamada: Data curation; writing – review and editing. Yusuke Sato: Data curation; writing – review and editing. Daisuke Yamada: Data curation; writing – review and editing. Haruki Kume: Project administration; supervision; writing – review and editing.

## Conflict of interest

The authors declare no conflict of interest.

## Approval of the research protocol by an Institutional Reviewer Board

Not applicable.

## Informed consent

Not applicable.

## Registry and the registration no. of the study/trial

Not applicable.
